# Correlation between elderly patients with COPD and the impact on immunity in tuberculosis patients: A retrospective study

**DOI:** 10.1097/MD.0000000000040140

**Published:** 2024-10-18

**Authors:** Yao Zhang, Yaping Zhang, Nanlan Ma, Zehui Huang

**Affiliations:** a Department of Tuberculosis, The Second Hospital of Nanjing, Affiliated to Nanjing University of Chinese Medicine, Nanjing, China; b General Medicine Department, Affiliated Central Hospital of Jiangnan University, Wuxi, China.

**Keywords:** comorbidity, COPD, immunological evaluation, lymphocyte subsets, tuberculosis

## Abstract

The prevalence of chronic obstructive pulmonary disease (COPD) and tuberculosis (TB) is increasing globally, yet their comprehensive impact on the immune system remains underexplored. This study aimed to provide a thorough assessment of the immune status of patients with COPD and tuberculosis (TB-COPD), including their pulmonary conditions, immune cell responses, and changes in lymphocyte subpopulations. A total of 151 patients with TB-COPD patients were included, and clinical data were compared between the TB-COPD group and a group of TB patients without COPD (TB-NCOPD). Lung imaging findings and peripheral blood immune cell levels were compared between the 2 groups. Flow cytometry was used to analyze the absolute counts of lymphocyte subpopulations. The incidence of pulmonary lobe lesions and cavitation in the TB-COPD group aged 70 years or older was significantly higher than that in the control group. At the immune cell level, patients with TB-COPD showed a significant reduction in total lymphocytes, CD4+ T lymphocytes and CD4+/CD8+ ratio. Regardless of COPD status, the CD4+ T cell count in the CMV-infected group was significantly lower than that in the uninfected group (*P* < .05). Additionally, the CD4+/CD8+ ratio in the COPD + TB CMV + group was significantly lower than that in the uninfected group. Analysis of lymphocyte subpopulations revealed a decrease in the counts of CD4+ T lymphocytes in patients with TB-COPD, potentially associated with the chronic inflammatory state induced by COPD. The one-month treatment outcomes showed that the improvement rate in the control group was 70.58%, which was significantly higher than the 38.92% in the COPD + TB group (*P* < .001). We observed a significant increase in the number of pulmonary cavity patients in the TB-COPD group, suggesting that COPD may be a potential risk factor for the formation of pulmonary cavities in patients with TB. At the immune cell level, TB-COPD patients showed a notable decrease in lymphocytes and CD4+ T lymphocytes, implying that COPD combined with pulmonary TB may significantly affect the immune system, leading to a reduction in the counts of key immune cells.

## 1. Introduction

Chronic obstructive pulmonary disease (COPD) is a prevalent condition worldwide^[1,2]^ and is characterized by chronic airflow limitation often accompanied by emphysema. Its prevalence has increased over the past decade.^[3]^ making it the third leading cause of death globally as of 2020.^[4]^ COPD predominantly affects older individuals and is intricately linked to the aging process, particularly with the dysfunction of the immune system.

Aging leads to significant alterations in the immune system, with immunosenescence emerging as a key factor.^[5,6]^ This process involves a reduction in the number and function of immune cells, impaired immune responses, and dysregulation of immune mechanisms.^[7]^ The immune responses constitutes an extraordinary defense network comprising tissues, cells, and molecules that play a vital role in protecting the body against pathogens such as bacteria, viruses, and other potential threats. However, as individuals age, this once vigilant guardian undergoes transformations, rendering the host more susceptible to infections, weakening its ability to mount effective defenses against new threats, and exacerbating the risk of various age related diseases, including cancer, cardiovascular diseases, and tuberculosis (TB).^[8–10]^

Furthermore, a notable feature of immunosenescence is its dynamic interaction with cytomegalovirus (CMV), a prevalent human pathogen. Researchers have long been investigating the role of CMV in shaping immune responses and posing questions about its contributions to the aging process and age-related diseases. The presence of CMV infection, which is prevalent in older adults, may exacerbate immunosenescence, further complicating the relationship between COPD, immunosenescence, and TB infection.

The pathogenesis of pulmonary TB is quite complex.^[11]^ Existing research indicates that, in addition to the toxicity and quantity of Mycobacterium TB, compromised immune function plays a crucial role in the development of TB. Immunodeficiency is also recognized as a significant risk factors for the occurrence of pulmonary TB.^[12]^ In the elderly population, TB exhibits specific characteristics in terms of incidence and treatment complexity, including the ambiguity of typical symptoms, uncertainty in disease progression, and difficulties in diagnosis. Compared to younger patients, TB in the elderly may manifest atypically, leading to potential oversight or misdiagnosis. Therefore, immune dysregulation in the COPD may also significantly impact the control of TB infection. As the immune system ages, older COPD individuals may face increased challenges in combating Mycobacterium TB, resulting in an elevated risk of infection.

Despite the growing recognition of COPD as a major public health issue, particularly among the elderly, the correlation between COPD and TB remains relatively understudied. This retrospective study aimed to investigate the relationship between COPD in elderly patients and their susceptibility to TB infection, focusing on the impact of COPD on immune function and TB outcomes. Through a retrospective analysis of patient records, we sought to elucidate the association between COPD and TB incidence, disease severity, and treatment outcomes in the elderly populations.

## 2. Patients and methods

### 2.1. Study design and population

In this retrospective study, we included 306 patients admitted to the Department of TB, The Second Hospital of Nanjing (Nanjing, China) from May 2022 to December 2023, with a focus on pulmonary TB-COPD.

### 2.2. Study participants

#### 2.2.1. Inclusion criteria

Elderly individuals aged ≥ 65 years.

Patients diagnosed with both COPD and pulmonary TB. The diagnosis of COPD followed the guidelines for the diagnosis and Treatment of Chronic Obstructive Pulmonary Disease, and pulmonary TB diagnosis followed the guidelines for the diagnosis and classification of TB. Patients not receiving glucocorticoid or immunosuppressive therapy. Patients with stable vital signs and illness duration of more than 3 months were included in the experimental group. Meanwhile, Elderly patients who met the diagnostic criteria for pulmonary TB but without COPD were assigned as the control group.

#### 2.2.2. Exclusion criteria

Patients concurrently experience other significant complications or diseases are excluded, such as autoimmune diseases, granulocytopenia, severe hepatic or renal diseases, malignancies, diabeted, cardiovascular, HIV infection, and pregnancy. Patients treated with glucocorticoids or immunosuppressants.

### 2.3. The diagnostic criteria for COPD

Patients with symptoms such as chronic cough, sputum production, and breathlessness undergo pulmonary function tests. The results indicate an FEV1/FVC ratio of < 0.7 after inhalation of bronchodilators, indicating the presence of incomplete reversible airflow obstruction, which is a necessary criterion for diagnosing COPD.^[13]^

#### 2.3.1. The severity of COPD can be classified into 4 stages

Mild (Gold I): Airflow limitation is mild and often accompanied by chronic cough and sputum production. The FEV1/FVC ratio was less than 70%, and FEV1 was greater than or equal to 80% of the predicted value.

Moderate (Gold II): Airflow limitation worsens, leading to breathlessness. The FEV1/FVC ratio was < 70%, with an FEV1 between 50% and 79% of the predicted value.

Severe (Gold III): The airflow limitation is very pronounced, with severe breathlessness affecting daily activities. The FEV1/FVC ratio was < 70%, with an FEV1 between 30% and 49% of the predicted value.

### 2.4. The diagnostic criteria for TB

Clinical Evaluation: Patients presenting with symptoms suggestive of pulmonary tuberculosis, such as chronic cough, hemoptysis, chest pain, and unexplained weight loss, underwent comprehensive assessment by experienced clinicians.

Radiological Imaging: Chest X-rays or computed tomography (CT) scans were performed to evaluate the presence of characteristic pulmonary tuberculosis lesions, such as cavities, infiltrates, and opacities.^[14]^

Microbiological Examination: Suspected pulmonary tuberculosis patients provided sputum samples for microbiological examination. Acid-fast bacilli (AFB) smear microscopy and/or GeneXpert MTB/RIF assay were conducted to detect the presence of Mycobacterium tuberculosis (MTB) in sputum samples.^[15]^

Laboratory Testing: In addition to microbiological examination, laboratory tests such as sputum culture and tuberculosis-specific serological tests (interferon-gamma release assay) were performed to confirm the diagnosis of pulmonary tuberculosis infection.

### 2.5. Peripheral blood collection, CD4/CD8 lymphocyte and CMV testing

Peripheral blood mononuclear cells (PBMCs) were isolated from 2 to 3 mL of peripheral blood samples collected from each patient. The PBMCs were then stained specific antibodies for flow cytometry analysis.

We used the following monoclonal antibodies from BD Biosciences: We used the following monoclonal antibodies from BD Biosciences: CD3-FITC (clone UCHT1, Cat No. 555332), CD16+ CD56-PE (clone B73.1, Cat No. 555516), CD8-PE (clone RPA-T8, Cat No. 555367), CD45-PerCP (clone 2D1, Cat No. 345809), CD4-APC (clone RPA-T4, Cat No. 555349), and CMV IgG antibodies (clone F83-2C8, Cat No. 340499t). Each antibody was used at a volume of 20 μL per sample. After the addition of antibodies, samples were incubated for 15 minutes at room temperature in the dark. Following this, 50 μL of the sample was added to each tube and mixed on a shaker for uniform distribution, then incubated in the dark for another 15 minutes. Next, Two milliliters of hemolysin (Cat No. 349202; BD Biosciences, San Jose, CA) were added to each sample, mixed thoroughly, and incubated for 10 minutes in the dark. After centrifugation at 500× g for 5 minutes, the supernatant was discarded. The cell pellet was then washed twice with 2 mL of phosphate-buffered saline (PBS) (BD Biosciences, Cat No. 55214), each time centrifuging at 500× g for 5 minutes. After the final wash, 100 μL of PBS was added to resuspend the cells.

The flow cytometric analysis was performed using a BD FACSCalibur flow cytometer (BD Biosciences). The absolute counts of CD4+ and CD8+ T cells were determined using BD TruCount™ tubes (BD Biosciences, Cat No. 340334), which contain a defined number of fluorescent beads for accurate absolute quantification. The analysis was conducted using BD CellQuest Pro software, which allowed for gating of lymphocyte populations based on forward and side scatter profiles. Lymphocyte subpopulations were then further gated using CD3 and CD45 expression.

The absolute counts of CD4+ and CD8+ T cells were calculated by correlating the number of gated events to the known number of beads in each sample, ensuring precise quantification.

### 2.6. Cytometry software data collection

Patient Demographics: Basic information was collected from the elderly participants in the study, including age, sex, duration of illness, and number of lesions.

Simultaneously, record patients’ laboratory indicators, including complete blood count, white blood cell count, monocyte count, lymphocyte count, neutrophil count, platelet count, and hemoglobin level. Routine biochemical indicators: albumin, globulin, A/G ratio.

Liver function tests included serum aspartate aminotransferase, serum alanine aminotransferase, lactate dehydrogenase, total bilirubin, and direct bilirubin. Information on geriatric diseases: Document on whether each patient had COPD. Information on tuberculosis diagnosis: Record whether each patient has pulmonary tuberculosis, including sputum smear examination, and sputum culture.

### 2.7. Observation of treatment outcome indicators

Lesion absorption was evaluated using chest CT scans performed both before and after 1 month of treatment. The chest CT scans were used to observe changes in the size, number, and characteristics of pulmonary lesions. Lesions were categorized as absorbed if significant reduction in size or complete resolution was observed compared to the baseline CT scan. The presence or absence of cavitation and other pathological features were also assessed to evaluate treatment response.

### 2.8. Ethics approval and consent to participate

The study was received by the Ethics Committee of the Second Hospital of Nanjing (Ethics Approval Number: 2024-LS-ky002) and conducted in accordance with the Declaration of Helsinki (Ethical Principles for Medical Research Involving Human Subjects). All patients who participated in the study have provided informed consent, and detailed informed consent forms were signed and documented.

### 2.9. Statistical methods

All statistical analyses were conducted using SPSS (version 22.0) and R (version 4.0.3). Based on normality, either the independent samples *t* test or Mann–Whitney *U* test was used to analyze measurement data for single-factor, two-level designs. Continuous data are presented as the mean ± standard deviation (M ± SD), and count data are expressed as [n (%)], with comparisons conducted using the χ^2^ test. Non-parametric data were presented as median and interquartile range. Depending on the sample size, the chi-square test or Fisher’s exact test was employed to compare nominal and ordinal variables. Principal component analysis (PCA) was performed using the prcomp function in R. A two-sided *P* < .05 was considered statistically significant.

## 3. Results

### 3.1. Patient characteristics

This study included 306 cases of tuberculosis (198 males and 108 females): patients with TB without COPD (TB-NCOPD) (n = 155) and with COPD (TB-COPD) (n = 151). The clinical characteristics of the TB-COPD group and TB-NCOPD group are presented in Table 1.

**Table 1:** Clinical characteristics of the study participants (n = 306).

Variables	Total	TB-NCOPD	TB-COPD	*P*
	(n = 306)	(n = 155)	(n = 151)	
Gender, n (%)				.668
Female	108 (35.29)	57 (36.77)	51 (33.77)	
Male	198 (64.71)	98 (63.23)	100 (66.23)	
Age, (yr)	71 (68, 76)	70 (67, 74)	72 (68, 79)	.055
Blood platelet count (BPC, 10^9^/L)	211.5 (158.25, 263.5)	200 (154, 255.5)	213 (162, 276)	.198
Hemoglobin (g/L)	118.62 ± 17.24	119.3 ± 17.12	117.92 ± 17.39	.484
Biochemical indexes				
Albumin (g/L)	36.35 (32.52, 39.18)	36.7 (33.2, 39.25)	35.7 (31.4, 39.1)	.135
Globulin (g/L)	27.95 (24.33, 31.6)	27.6 (24, 31.6)	28.1 (24.75, 31.6)	.608
A/G (ratio)	1.31 ± .34	1.34 ± .35	1.28 ± .33	.123
Liver function				
Serum glutamic oxaloacetic transaminase (SGOT, U/L)	21.1 (17.2, 27.1)	21.5 (18.2, 27.1)	2.5 (16.55, 27.65)	.163
Serum glutamic pyruvic transaminase (SGPT, U/L)	14 (1.12, 22.92)	13.9 (1.25, 21.45)	14.3 (9.75, 24.35)	.886
Lactic acid dehydrogenase (LDH, U/L)	190 (166, 216)	189 (169, 214)	190 (166, 22.5)	.839
Total bilirubin (TBiL, µmol/L)	12.2 (8.22, 17.62)	12.6 (8.2, 18.65)	12 (8.35, 16.8)	.404
Direct bilirubin (DBiL, µmol/L)	4.2 (2.62, 6.2)	4.4 (2.6, 6.2)	4.1 (2.7, 6.15)	.92
CMV status				
CMV seropositive	120 (39.22)	50 (32.26)	70 (46.36)	.03
CMV seronegative	186 (60.78)	105 (67.74)	81 (53.64)	

The data was showed as (M ± SD), and A significance level of *P* < .05 was employed to determine statistical significance.

CMV = cytomegalovirus, COPD = chronic obstructive pulmonary disease, TB = tuberculosis, TB-COPD = TB patients with chronic obstructive pulmonary disease, TB-NCOPD = TB patients without chronic obstructive pulmonary disease.

### 3.2. Changes in peripheral blood T-cell subsets among groups

The results of the median and interquartile range for CD4+ and CD8+ T cell counts in the overall population indicated that the level of CD4+ T cells (*P* = .002) and CD4+/CD8+ (*P* = .027) was significantly lower in patients with TB-COPD compared to those with TB-NCOPD. The level of CD8+ level was lower in patients with TB-COPD compared to those with TB-NCOPD, but the difference was not statistically significant (Table 2).

**Table 2:** Changes in peripheral blood T cell subsets among groups (Median, IQR).

Variables	Total	TB-NCOPD	TB-COPD	*P*
	(n = 306)	(n = 155)	(n = 151)	
CD4+	462 (316, 645.75)	487 (382.5, 686)	437 (268, 601)	.002
CD8+	259 (156.5, 379.25)	278 (178.5, 417.5)	234 (146.5, 357)	.095
CD4+/CD8+	2.02(1.08,2.57)	2.29(1.68,3.07)	2.06(1.06,3.11)	.027

IQR = interquartile range.

### 3.3. Changes in white blood cell subpopulations in blood tests

Table 3 summarizes the changes in white blood cell subpopulations observed in the blood tests, represented by the median values and IQR. The white blood cell count (WBC) and monocyte count (AMC) showed an increasing in the TB-COPD group compared to the control group, but the difference was not statistically significant. However, the lymphocyte count (ALC) was significantly elevated in the TB-COPD group, with statistical significance (*P* = .038) (Table 3).

**Table 3:** Changes in white blood cell subpopulations in blood tests (Median, IQR).

Variables	Total	TB-NCOPD	TB-COPD	*P*
	(n = 306)	(n = 155)	(n = 151)	
White blood cell count (WBC,10^9^/L)	5.97 (4.76, 7.46)	5.71 (4.54, 7.45)	6.28 (5.08, 7.46)	.086
Monocyte count (AMC, 10^9^/L)	0.54 (0.41, 0.71)	0.51 (0.39, 0.7)	0.56 (0.44, 0.72)	.167
Lymphocyte count (ALC, 10^9^/L)	1.18 (0.85, 1.62)	1.25 (0.93, 1.65)	1.08 (0.76, 1.6)	.038

### 3.4. The relationship between CMV status and immunosenescence

**Figure 1.**
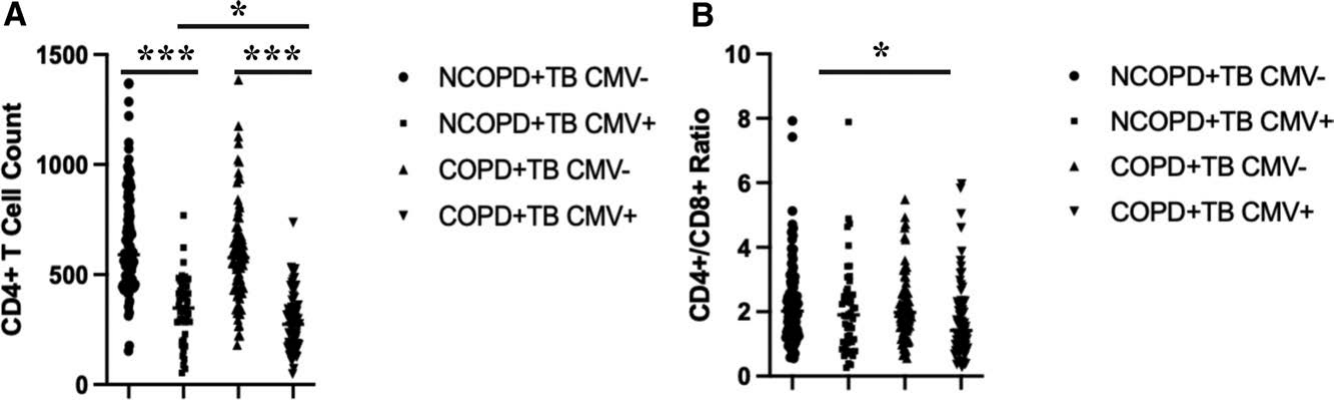
Impact of CMV Status on CD4+ T Cell Count and CD4+/CD8+ Ratio in TB Patients with or without COPD. Comparison of CD4+ T cell count among 4 groups: NCOPD + TB CMV−, NCOPD + TB CMV+, COPD + TB CMV−, and COPD + TB CMV+ (A). Comparison of CD4+/CD8+ ratio among the same 4 groups (B).

Based on differences in COPD, TB, and CMV infection status, the results show that the CD4+ T cell count in the CMV-infected groups, regardless of COPD status, is significantly lower than in the CMV-uninfected groups (Fig. 1A). Additionally, the CD4+/CD8+ ratio in the COPD + TB CMV + group is significantly lower than in the CMV-uninfected groups, with the difference being statistically significant, marked as * (*P* < .05) (Fig. 1B).

### 3.5. Comparison of age, immune response, and pulmonary lesions in patients with TB with or without COPD

It is widely recognized that age is associated with immune response in patients with TB and COPD or its complications. The elderly experience a significant decline in immune function, making them more susceptible to Mycobacterium tuberculosis infection during COPD, leading to poorer treatment outcomes.

In patients with TB accompanied by or without COPD, we observe differences in age distribution, immune response, and severity of pulmonary lesions. Patients aged 70 years and above tend to exhibit more severe pulmonary lesions compared to those under 70, accompanied by a corresponding decline in immune response. As shown in Table 4, the number of patients with pulmonary lobe lesions and cavities in the ≥ 70 years TB-COPD group is significantly higher than in the < 70 years TB-NCOPD group. Additionally, the TB-DM group shows a significantly higher number of patients with pulmonary lobe lesions and cavities compared to the TB-NCOPD group.

**Table 4:** The relationship between age and pulmonary lesions.

	TB-NCOPD (n = 155)		Total	TB-COPD (n = 151)		Total
	Age < 70 years (n = 85)	Age ≥ 70 years (n = 70)		Age < 70 years (n = 65)	Age ≥ 70 years (n = 86)	
Pulmonary lobe lesion						
Mild (1–2 lobes) (%)	52 (61.18)	38 (54.29)	90	27 (41.54)	19 (22.09)	46
Severe (3–5 lobes) (%)	33 (38.82)	32 (45.71)	65	38 (58.46)	67 (77.91)	105
Cavity						
No (%)	40 (47.06)	30 (42.86)	70	25 (38.46)	34 (39.53)	59
Yes (%)	45 (52.94)	40 (57.14)	85	40 (61.54)	52 (60.47)	92

### 3.6. Impact of various measurements on TB-COPD patients

**Figure 2.**
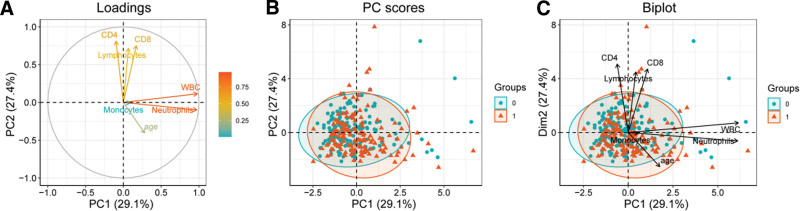
Principal Component Analysis (PCA): PCA1 axis and PCA2 axis explained 29.1% and 27.4% of the overall variation in the data (A). In the PC scores plot, the data from TB-COPD patients exhibited significance. (B) Using the fviz_pca_ind function for visualization, it illustrates the distribution of each sample(C). The arrow colors represent the contributions of each variable to PCA. The angles between arrows indicate the relationships between variables: acute angles represent positive correlations, obtuse angles represent negative correlations, and right angles represent no correlation.

The influence of different measurements on patients with TB-COPD patients was assessed using PCA, including CD4, CD8, monocyte, lymphocyte, neutrophil, age, and WBC counts. The explanatory percentages for PCA axes 1 and axis 2 were 29.1% and 27.4%, respectively. The combined contribution of these 2 axes explained more than 50% of the overall data variability, indicating that the first 2 axes of PCA could effectively capture the fundamental patterns in the data. Notably, WBC and neutrophil made the greatest contributions to PCA axis 1, while CD4, CD8, and lymphocyte had the highest contributions to PCA axis 2 (Fig. 2A). The PCA score plot demonstrated significant distinctions in the data of TB-COPD patients (Fig. 2B). Additionally, a biplot was employed to illustrate loadings and PC scores (Fig. 2C).

### 3.7. Impact of COPD on innate and adaptive immunity in TB

**Figure 3.**
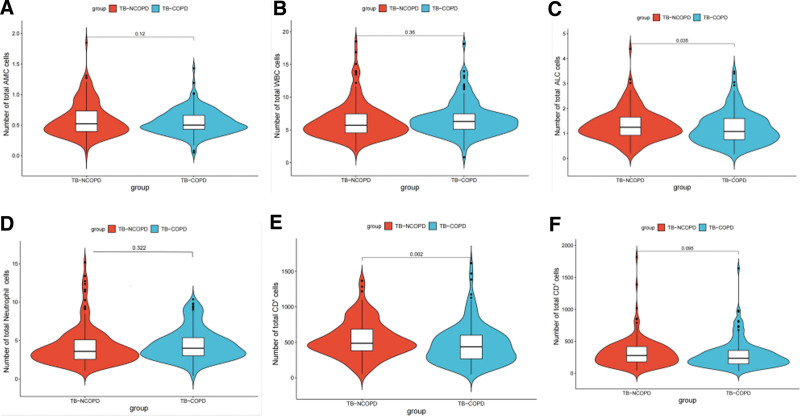
The comparison of lymphocyte subsets between TB-COPD and TB-NCOPD patients. The absolute counts of AMC cells (A), WBC cells (B), ALC cells (C), Neutrophil (D), CD4 T lymphocytes (E), and CD8 T lymphocytes (F) between TB-NCOPD and TB-COPD patients.

The absolute counts of ALC (*P* = .036) (Fig. 3C) and CD4 T lymphocytes (*P* = .002) (Fig. 3E) were significantly higher in TB-NCOPD patients compared to TB-COPD patients. Additionally, there was no statistically significant difference in the total counts of AMC, Neutrophil, WBC, and CD8 T lymphocytes between TB-NCOPD and TB-COPD patients. Interestingly (Fig. 3A, B, D, and F), TB-COPD patients exhibited a decreasing trend in the total counts of AMC, Neutrophil, WBC, and CD8 T lymphocytes compared to TB-NCOPD patients.

### 3.8. The relationship between CD4+ levels and the severity of TB

**Figure 4.**
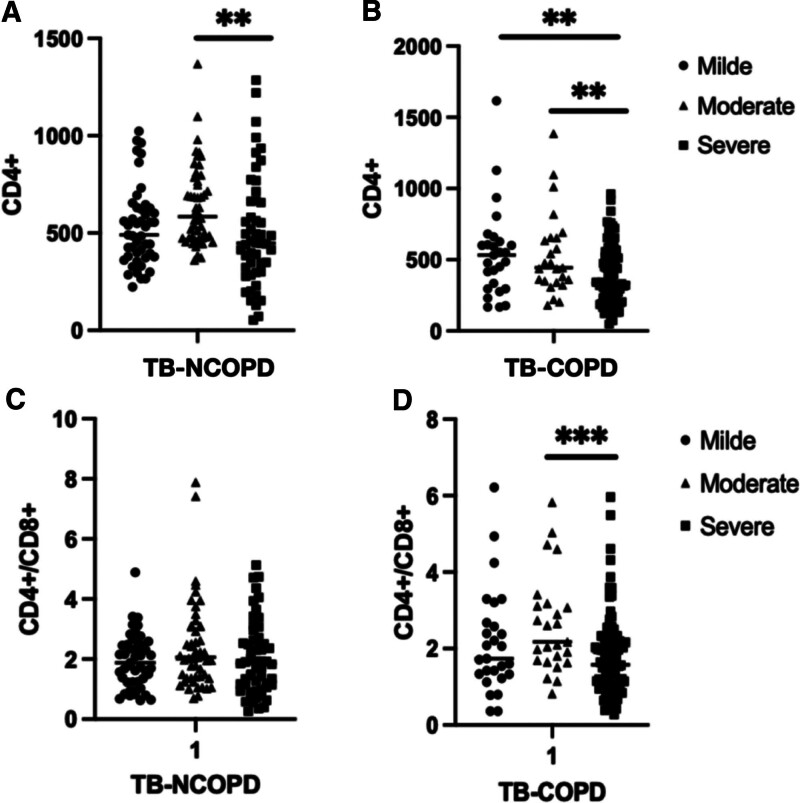
The relationship between CD4+ levels and the severity of TB. Comparison among 3 different levels of TB-NCOPD (A), Comparison among 3 different levels of TB-COPD (B). The relationship between CD4+/CD8+ levels and the severity of TB. Comparison among 3 different levels of TB-NCOPD (C), Comparison among 3 different levels of TB-COPD (D).

Based on the nature and extent of lesions observed on chest imaging, patients with TB were classified into 3 groups: mild, moderate, and severe. Mild: Lesions were confined to a single lung lobe, without cavitation (47 cases without COPD, 27 cases with COPD); Moderate: Lesions involved 2 or more lung lobes on 1 side, with cavitation if present, totaling less than 4 cm in diameter (51 cases without COPD, 25 cases with COPD); Severe: Lesions involved multiple lung lobes on both sides, with multiple cavitations (55 cases without COPD, 97 cases with COPD). In the TB-COPD group, serum cytokine CD4+ levels were significantly decreased in the moderate and severe groups; compared to the mild and moderate groups and CD4+ levels were decreased in the severe group (*P* < .001) (Fig. 4A and B). In the TB-COPD group, the serum cytokine CD4+/CD8+ levels were significantly lower in the severe group compared to the mild and moderate groups (*P* < .001) (Fig. 4C and D).

### 3.9. Comparison of treatment outcome indicators between the 2 groups of patients

compares the one-month treatment effectiveness between the Control Group and the COPD + TB Group. The Control Group had a higher efficiency rate of 70.58%, with 108 patients showing improvement out of 155 total. In contrast, the COPD + TB Group had a significantly lower efficiency rate of 38.92%, with only 58 patients improving out of 151 total. A Fisher’s exact test was used to evaluate the statistical significance of the difference between the 2 groups. The result yielded a *P* < .001 (Table 5).

**Table 5:** The comparison of 1-month treatment effectiveness between the 2 groups.

Group	Total	Improved	Not improved	Efficiency rate
Control Group	155	108	47	70.58
COPD + TB	151	58	93	38.92
χ^2^				–
*P*				<.001

“–” indicates Fisher’s exact test was used, with no exact value available.

## 4. Discussion

This study aimed to gain a comprehensive understanding of the impact of COPD on the immune system of patients with TB, with a particular focus on immune responses, and changes in lymphocyte subpopulations.^[16]^ In recent years, the incidence of COPD and TB patients has been increasing annually, both of which are common diseases in the elderly population. It is well known that COPD, as a disease prevalent in the elderly, can significantly affect lung health and immune function. The immumosenescent individuals often undergoes aging with advancing age, a process closely associated with diminished responses to chronic diseases, infections, and viral control.^[17–19]^ Characteristics of immumosenescent include the presence of chronic inflammation, poor control of viral infections, and changes in the T cell repertoire. With increasing age, levels of inflammatory markers in the immune system tend to rise, along with an increased ratio of CD8+ (effector T cells) to CD4+ T (helper T cells) cells. Concurrently, there is a reduction in the pool of naïve T cells (comprising both CD4+ and CD8+ T cells), which may be linked to age-related declines in immune function. This results in a relative increase in the proportion of memory T cells compared to naïve T cells.^[20]^

Our study revealed a significant reduction in CD4+ T cells in TB-COPD patients, accompanied by a decrease in the CD4+/CD8+ ratio, indicating severe cellular immune dysfunction in these patients. Additionally, we observed a notable increase in the formation of pulmonary cavities in TB-COPD patients, suggesting that COPD may accelerate TB-induced lung damage. Consistent with existing literature, our findings further underscore the critical role of chronic inflammation and immunosenescence in the development of immune dysfunction.

Furthermore, reactivation of chronic viral infections may also expedite the process of immune aging, with CMV reactivation recognized as a primary driver of immune senescence. CMV belongs to the herpesvirus family and can cause cytomegalovirus infection. In most cases, CMV infection is asymptomatic, but it can lead to severe complications in individuals with impaired immune function. In immunocompetent individuals, CMV infection is typically controlled by the immune system, but the virus persists and can reactivate when immunity is compromised.^[21]^ The results of this study showed that, regardless of whether the patients had COPD, the CD4+ T cell count in the CMV-infected group was significantly lower than that in the uninfected group. CMV reactivation leads to CD4+ T cell depletion, thereby weakening the immune system’s ability to respond to other infections. Additionally, the CD4+/CD8+ ratio in the COPD + TB CMV-infected group was significantly lower than that in the uninfected group, suggesting that CMV infection may further exacerbate immune function decline. This indicates that CMV reactivation in patients with coexisting COPD and TB may have a more profound negative impact on the immune system, particularly affecting CD4+ T cell levels and immune balance. Although CMV infection typically leads to CD8+ T cell expansion, the function of CD8+ T cells may be suppressed or exhausted due to the long-term pressures of chronic inflammation, COPD, and TB on the immune system. This means that even with viral reactivation, the immune system may no longer effectively stimulate CD8+ T cell expansion.

The coexistence of COPD and TB may further exacerbate immune dysregulation, impair immune function, and lead to alterations in T cell subset differentiation. Earlier studies have indicated that tuberculosis is not only an infectious disease but also exhibits characteristics of immune dysregulation.^[22]^ The interaction between Mycobacterium tuberculosis invasion and the dysregulation of the host immune system is crucial for the progression from latent tuberculosis infection to active tuberculosis.^[23]^

The role of immune function in susceptibility to tuberculosis infection among COPD patients is crucial. In this study, we conducted subgroup analysis based on age, dividing patients into < 70 years old and ≥ 70 years old. The results indicate that the number of patients with pulmonary lobe lesions and cavities in the ≥ 70 years TB-COPD group is significantly higher than in the < 70 years TB-NCOPD group. Additionally, the TB-DM group shows a significantly higher number of patients with pulmonary lobe lesions and cavities compared to the TB-NCOPD group.

Immune dysfunction plays a crucial role in the susceptibility of COPD patients to coexisting pulmonary TB.^[24]^ We examined the immune responses between TB-NCOPD patients and TB-DM patients. The results revealed that the number of CD4+ T cells (*P* = .002) and CD4+/CD8+ (*P* = .027) in the TB-COPD group was significantly lower than that in the TB-NCOPD control group. Additionally, the count of ALC cells was significantly reduced in the TB-COPD group (*P* = .038). Studies have suggested that abnormalities in peripheral blood T lymphocytes may be associated with the pathogenesis of airflow limitation. COPD itself is a chronic inflammatory disease characterized by persistent inflammation in the airways and lung tissues.^[25]^ This chronic inflammation may lead to sustained activation of the immune system, ultimately resulting in immune exhaustion and functional impairment.^[26,27]^ Our findings indicate cellular immune impairment in tuberculosis patients. Moreover, the degree of cellular immune dysfunction is more severe in patients with pulmonary TB combined with COPD than in those with pulmonary tuberculosis alone.

According to reports, CD4 play a crucial role in combating Mycobacterium tuberculosis infection.^[28]^ We focused on the impact of COPD on lymphocyte subpopulations. The study results indicated a significant reduction in the counts of CD4 in TB-COPD patients, possibly related to the chronic inflammatory state induced by COPD.

This is consistent with other research findings, indicating that the extent of cellular immune impairment in TB patients with coexisting COPD is more severe than in patients with pulmonary tuberculosis alone or those with COPD alone.^[29]^ Chronic inflammation is common in COPD patients and may affect the generation, proliferation, and function of lymphocytes, suggesting that COPD might exacerbate immune cell apoptosis or inhibit their normal function, contributing to the immune depletion observed in TB patients.^[30]^

Secondly, we considered the impact of COPD on AMC, Neutrophil, WBC,^[31]^ which play a crucial role in immune defense, particularly in infection and inflammatory responses. We observed a decrease trend in the number of AMC, Neutrophils, WBC in TB-COPD patients, suggesting that COPD may inhibit these important immune cells, weakening their ability to clear Mycobacterium tuberculosis. This is a significant concern for TB patients, as compromised AMC, Neutrophil, WBC function may accelerate the progression and complications of tuberculosis. Furthermore, the significant increase in the number of pulmonary cavity patients indicates that COPD may accelerate pathological changes in the lungs of TB patients.^[32]^ This could be related to impaired lung ventilation, chronic inflammation, and decreased lung immune function caused by COPD. TB patients are already prone to developing pulmonary cavities, and the addition of COPD may act as an accelerator in this process.

The results of PCA indicate that WBC and neutrophils contribute most significantly to the primary PCA axis, suggesting that inflammation and immune response play key roles in TB-COPD patients. WBC and neutrophils are the most common immune cells in the body, and their increase may reflect a state of chronic inflammation and activation of the immune system, consistent with the common immune dysregulation and persistent inflammatory responses observed in COPD and TB patients.

On the other hand, CD4, CD8, and lymphocytes contribute more significantly to the second axis of PCA, which may be related to immune function and cell-mediated immune responses. CD4 and CD8 T lymphocytes are crucial components of the immune system and play key roles in coordinating immune responses. CD4+ T cells are involved in coordinating immune responses, promoting B cell activation, and regulating cell-mediated immune responses, while CD8+ T cells primarily participate in cell-mediated immune responses by eliminating infected cells.

Previous studies have also shown an association between cytokine levels and the severity of pulmonary tuberculosis. Cytokines such as IL-1, sIL-2R, TNF-α, IL-5, IL-6, and IFN-γ play important roles in pulmonary tuberculosis patients, and their elevated levels may be associated with disease severity and inflammatory status. Additionally, nutritional levels and inhaled corticosteroids are closely related to the development and severity of tuberculosis, which may affect disease progression by influencing immune responses and inflammatory levels.In conclusion, the impact of COPD on the immune system of tuberculosis patients extends beyond a reduction in specific immune cell counts and involves inflammation and lung damage. These findings provide valuable insights for future research into the reciprocal relationship between COPD and tuberculosis, with the potential to improve treatment and management strategies for this specific patient population.

The study has several limitations. One of the limitations of this study is that it was a retrospective analysis conducted at a single hospital in China, which may limit the generalizability of the findings. The specific geographic region and demographic characteristics of the patients may not reflect those in other parts of the world, particularly in areas where healthcare practices, environmental factors, and disease prevalence differ. For instance, variations in COPD and TB management practices, access to healthcare, and environmental exposure could influence the outcomes. Additionally, the relatively small sample size could affect the reliability and generalizability of the results. Furthermore, the study is limited to observing outcomes without delving into the underlying biological mechanisms, potentially constraining the interpretation of the results.

## 5. Conclusion

In conclusion, this retrospective study found that COPD may be a potential risk factor for the formation of pulmonary cavities in tuberculosis patients. Additionally, the significant reduction in CD4+ T lymphocytes in TB-COPD patients indicates that the immune function of patients with coexisting COPD and tuberculosis is more severely impaired. Our findings also suggest that CMV reactivation may further exacerbate immune function decline in these patients. Future research should adopt longitudinal designs to further explore the long-term impact of COPD, CMV reactivation, and their interaction with tuberculosis on immune function, and to develop potential immune intervention strategies to improve clinical outcomes.

## Acknowledgments

All authors read and approved the final manuscript. We are grateful to all the researchers involved in this study.

## Author contributions

**Data curation:** Yao Zhang, Yaping Zhang, Nanlan Ma.

**Formal analysis:** Yao Zhang, Zehui Huang.

**Investigation:** Nanlan Ma.

**Methodology:** Yaping Zhang.

**Software:** Yaping Zhang.

**Writing – original draft:** Yao Zhang, Zehui Huang.

**Writing – review & editing:** Yao Zhang, Zehui Huang.
